# CURRICULUM FOR PRACTICE AND LEADERSHIP IN NURSING HOMES: THE US RESPONSE

**DOI:** 10.1093/geroni/igae098.0713

**Published:** 2024-12-31

**Authors:** Ann Kolanowski, JoAnne Reifsnyder, Jacqueline Dunbar-Jacob

**Affiliations:** The Pennsylvania State University, University Park, Pennsylvania, United States; University of Maryland Baltimore, Baltimore, Maryland, United States; University of Pittsburgh, Pittsburgh, Pennsylvania, United States

## Abstract

The Teaching Nursing Home (TNH), which began in the 1980s under the leadership of Dr. Mathy Mezey, was a visionary approach to improving the lives of older adults in nursing homes through the efforts of the interdisciplinary healthcare team. The project had extraordinary outcomes, but for a host of political and economic reasons was never brought to full realization. Forty years and a pandemic later, the need for improving quality care in our nations’ nursing homes took center stage. Dr. Terry Fulmer (John A Hartford Foundation) and Nancy Zionts (Jewish Healthcare Foundation) provided extraordinary leadership to address what is now widely regarded as a devastating gap in the US healthcare system. With over $5 million in funding, academic/practice partnerships were revisited in a pilot project with renewed energy, creativity, and resolve by three major universities in Pennsylvania and four affiliated nursing homes. Early on, the lack of curriculum models for faculty educating pre-licensure students in the delivery of quality care in nursing home settings was identified as a barrier to progress in the TNH project. The authors of this paper present the groundbreaking work that was done by an interdisciplinary panel of over 40 expert healthcare practitioners and academicians who came to consensus in the identification and qualitative development of a curriculum that prepares healthcare professionals and their faculty through academic/practice partnerships. Competencies include: creating a culture of care; improving diversity, equity and inclusion; person/family-centered care; models of care delivery; geriatric syndromes; interdisciplinary practice; regulation and finance; and information technology.

